# Spontaneous mammalian models for Alzheimer's disease and dementia

**DOI:** 10.1093/braincomms/fcaf287

**Published:** 2025-07-29

**Authors:** Madeleine Ford, Frank J Gunn-Moore, Mark P Dagleish

**Affiliations:** School of Biology, Biomedical Sciences Research Complex, University of St Andrews, St Andrews KY16 9ST, UK; School of Biology, Biomedical Sciences Research Complex, University of St Andrews, St Andrews KY16 9ST, UK; Division of Veterinary Pathology, Public Health and Disease Investigation, School of Biodiversity, One Health and Veterinary Medicine, University of Glasgow, Glasgow G61 1QH, UK

**Keywords:** models, mammalian, Alzheimer’s disease, non-laboratory

## Abstract

Globally, the human population is ageing, and, consequently, the prevalence of major neurocognitive disorders is increasing, resulting in a greater need for novel dementia therapeutic interventions. Animal models are invaluable in studying underlying pathological processes in human diseases and with evidence for rising life expectancy in many domesticated animals studies have investigated neurocognitive disorders in several non-human species. Rodents have been used extensively as animal models, but this review will examine published literature suggesting candidate non-laboratory animal models for studying dementia, especially human Alzheimer's disease. Comparison of the physiological, pathological and clinical features of companion animals, farm animals and marine mammals shows that although many animals develop amyloid plaques and, to lesser degree, hyperphosphorylated tau protein, very few develop neurofibrillary tangles or neuronal loss to the same extent as humans with Alzheimer's disease. Several hypotheses are proposed as to why, as yet, no animals have been found to spontaneously develop Alzheimer's disease-like pathology to the same level as humans but highlight specific aspects where these models may be useful if developed further.

## Teaser

No laboratory animal model fully reproduces Alzheimer's disease. However, companion (dogs, cats, horses), farm (cattle, sheep, pigs, donkeys) and marine mammals (odontocetes, pinnipeds) spontaneously develop amyloid plaques and, to lesser degree, hyperphosphorylated tau. Therefore, these species may be more representative models of specific aspects of Alzheimer's disease if developed further.

## Graphical Abstract

**Graphical Abstract:**
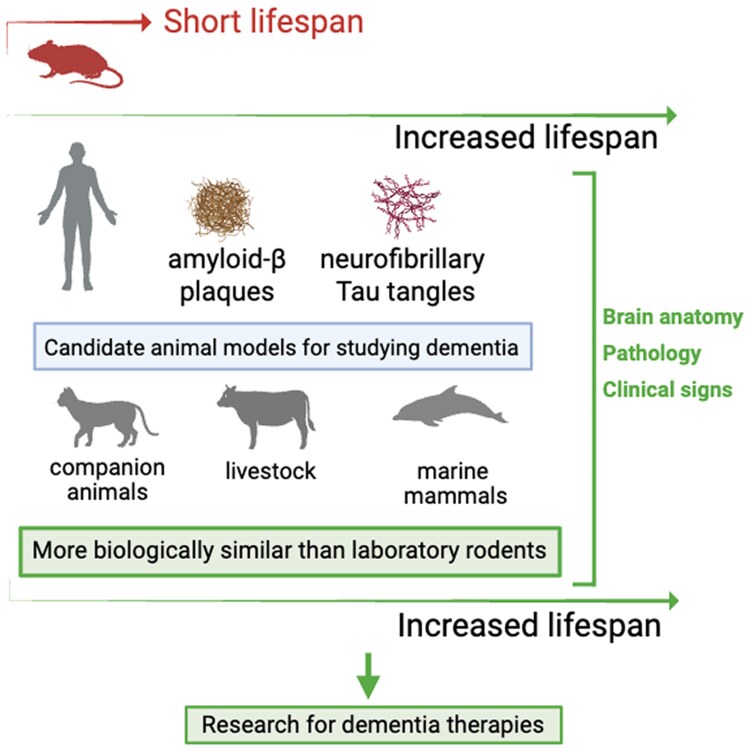

## Introduction

Human life expectancy is increasing due to improved health care with almost 900 million individuals now over 60 years old, and ageing remains the most significant risk factor for neurodegeneration—the progressive loss of neurons in the brain.^1^ With an ageing population, the number of individuals living with a neurodegenerative condition has increased significantly and will continue to increase.^2^ In the UK, an estimated 1 in 14 individuals over 65 years old, and a total of 55 million worldwide, live with dementia, and this is expected to nearly triple by 2050^2,3^ with ∼9.9 million new dementia cases expected every year.^1^

The brain is specifically vulnerable to ageing with, commonly, several processes and functions, such as thinking and memory, declining in older age.^1^ However, people with dementia experience severe cognitive and behavioural changes compared to healthy, ageing adults. Dementia refers to the interference of an individual's daily life, including the ability to remember objects and events, perform simple tasks, utilize speech, remain socially and spatially attentive and regulate emotions due to severe cognitive decline.^4,5^ Many diseases can cause dementia, Alzheimer's disease being the most common and causing approximately two-thirds (>35 million) of all diagnosed dementia cases, but it also includes vascular dementia, dementia with Lewy bodies and frontotemporal dementia.^3,6^

These distinct diseases are, typically, associated with lesions in the brain due to accumulation of pathological proteins (proteinopathy), such as an accumulation of amyloid-beta (Aβ) into plaques or tau proteins into neurofibrillary tangles (NFTs) in Alzheimer's disease, Aβ in vascular dementia, alpha-synuclein in dementia with Lewy bodies or TDP-43 protein in frontotemporal dementia, in the parenchyma and/or vasculature, or secondary to vascular disease and/or a stroke.^3,6,7^ Additionally, dementia sufferers often show a significant loss of brain neurons and brain atrophy. As age-related neuropathology varies between individuals,^6^ distinguishing between normal brain ageing and pathology is crucial. Understanding these pathological processes, the brain regions affected and how these result in clinical disease is critical to finding and developing therapeutics to reduce the impact of neurodegenerative diseases on the ageing population, for which presently there are no cures.^1^

Animal models are invaluable to human research, specifically in studying biological, physiological and pathological processes implicated in many human disorders.^8^ Many of these are based on models in rodents but these species are very short-lived compared to humans. Furthermore, human life expectancy has increased, reducing further the relevance of rodent models. However, the life expectancy of many domesticated animals has increased,^6^ and even though the presence of spontaneous neurodegeneration and subsequent cognitive decline in animals is unclear, a number of studies have identified both similarities and differences compared to humans. For example, relatively recent studies have shown comparable human-like neurodegenerative pathology akin to Alzheimer's disease changes in domesticated companion animals,^6,9-12^ livestock^13-15^ and marine mammals.^16-18^ In addition to pathology, some animals developed clinical signs similar to the social, physical and psychological symptoms experienced by human patients with dementia. As well as furthering the understanding of human cognition mechanisms underlying age-related pathology, studying animals’ behaviours, cognitive abilities and neuropathologies will expand knowledge of their individual cognitive and evolutionary processes.^13,19^

A review of human and animal cognition and associated dysfunction presents a significant research opportunity. Cognitive dysfunction syndrome defines cognitive deterioration in animals not caused by other diseases.^6^ However, evidence suggests that ‘dementia’, currently used only to describe human neurodegenerative diseases, should also include neurodegenerative diseases in other animals. This review will explore the evidence of spontaneous human dementia-like pathology and cognitive dysfunction in companion animals (cats, dogs and horses), livestock (donkeys, pigs, cattle and sheep) and marine mammals (dolphins, seals, sea lions and walruses). It is noteworthy that most of the studies have investigated the pathogenic changes associated with Alzheimer's disease, specifically changes in Aβ amyloid and tau. By defining animal cognitive dysfunction syndrome as dementia in a variety of species, we highlight priorities for future research to aid identifying therapies for human dementia.

## The human brain

The human brain is a complex organization of several distinct regions composed of nerve cells and blood vessels responsible for the chemical and electrical signals controlling various processes including thought, memory, emotion and motor function, as well as breathing, heart rate and pain perception.^20^

The longitudinal cerebral fissure divides the human cerebrum into the left and right hemispheres, each of which is further divided into four lobes: frontal, parietal, temporal and occipital.^21^ The hemispheres encompass the cerebral cortex, which receives, processes and interprets information from the five senses: vision, hearing, taste, smell and touch and, arguably, proprioception,^22^ and integrates these separate inputs in the cortex using neuronal networks between these lobes.^20^ Below the cerebral cortex lies the sub-cortex consisting of the basal ganglia, the limbic system (including the thalamus, hippocampus, amygdala and hypothalamus), the cerebellum and the brain stem, the latter continuous with the spinal cord.^20,21^ These brain regions are responsible for movement and posture, emotional processing and vision, amongst other physiological functions necessary for survival including controlling heart rate, blood pressure, sleep cycle and swallowing.^20^

## Animals—brains, pathology and behaviour

**Figure 1:**
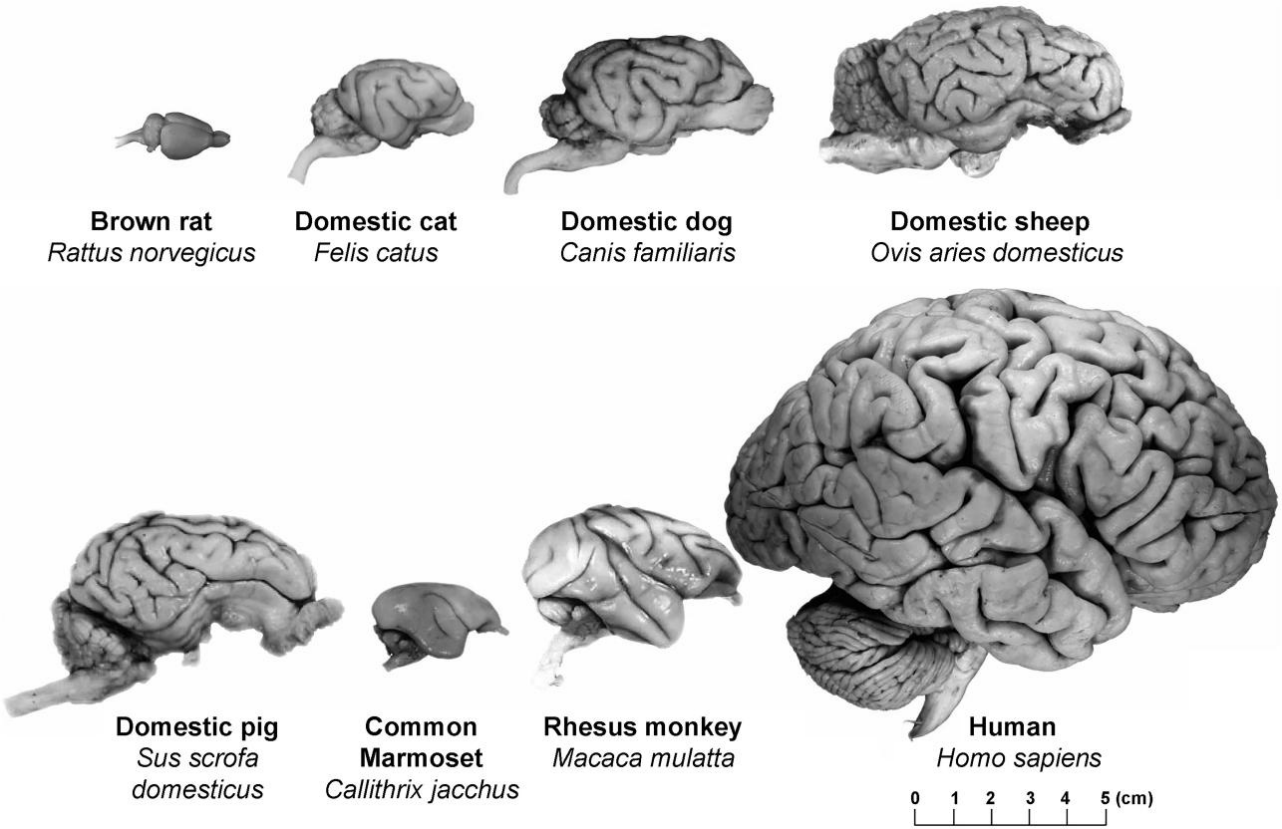
**Comparison of different species’ brains.** A visual representation of gyrencephalic brains and mean brain weight (g) in humans (1320), domestic cats (25.6), dogs (50–130), sheep (175), pigs (180) and the rhesus monkey (179) in comparison to the much smaller lissencephalic brain of a brown rat (1.9).^24,25^

Studies on ageing processes and neuropathology in animals with cognitive dysfunction syndrome have primarily focused on rodents and invertebrates in laboratory settings due to accessibility and controlled environments.^6,8^ However, larger animals with longer lifespans, such as cats, dogs, equids, pigs and marine mammals, appear to be more promising candidates.^9-18,23^ Additionally, larger mammal brains are gyrencephalic (Fig. 1)^24^ like human brains and have the same highly conserved Aβ peptide amino acid sequence as humans^13,25^ that rodents lack (Table 1).^6^ With respect to tau, many yet not all of the six recognized isoforms present in humans have also been reported in other mammals (Table 2),^16,26^ although similarities and differences have not yet been fully elucidated. However, much of the functional topography in animal brains is unclear, making healthy, and subsequently declining, cognition in live animal subjects challenging to understand.

**Table 1:** Amyloid Precursor Protein gene amino acid sequence, letters in bold denote sequence differences, containing the region for Aβ peptide in humans and non-humans^16,26-29^

Human	5′-DAEFRHDSGYEVHHQKLVFFAEDVGSNKGAIIGLMVGGVVIA-3′
Cat	5′-DAEFRH**E**SGYEVHHQKLVFFAEDVGSNKGAIIGLMVGGVVIA-3′
Dog	5′-DAEFRHDSGYEVHHQKLVFFAEDVGSNKGAIIGLMVGGVVIA-3′
Chimpanzee	5′-DAEFRHDSGYEVHHQKLVFFAEDVGSNKGAIIGLMVGGVVIA-3′
Monkey^a^	5′-DAEFRHDSGYEVHHQKLVFFAEDVGSNKGAIIGLMVGGVVIA-3′
Delphinid^b^	5′-DAEFRHDSGYEVHHQKLVFFAEDVGSNKGAIIGLMVGGVVIA-3′
Pinniped^c^	5′-DAEFRHDSGYEVHHQKLVFFAEDVGSNKGAIIGLMVGGVVIA-3′
Mouse	5′-DAEF**G**HDSG**E**EV**R**HQKLVFFAEDVGSNKGAIIGLMVGGVVIA-3′

^a^ *Macaca fascicularis*.

^b^ *Grampus griseus*, *Stenella coeruleoalba*, *Tursiops truncatus*.

^c^ *Phoca largha*, *Eumetopias jubatus*, *Zalophus californianus*, *Neophoca cinerea*, *Odobenus rosmarus*.

**Table 2:** Tau isoform expression reported in select mammals^16,26^

	Species							
Isoform	Human	Mouse	Cat	Dog	Sheep	Pig	Pinniped	Bovine
0N3R	+	−	+	−	−	−	−	−
1N3R	+	−	−	−	+	+	+	+
2N3R	+	−	+	+	+	+	+	+
0N4R	+	+	+	+	−	−	+	−
1N4R	+	+	+	+	+	+	+	+
2N4R	+	+	+	+	+	+	+	+

+ denotes tau isoform clearly present; − denotes tau isoform weakly detectable or absent.

## Companion animals

Companion animals are increasingly studied due to their coexistence with humans.^6,30^ As with humans, the lifespans of cats and dogs are increasing, leading to an increase in animals over 10 years old classed as ‘senior’.^31,32^ Age-related cognitive dysfunction syndrome in cats and dogs is widely acknowledged, affecting an estimated 28% of cats aged 11–14 years and dogs aged 11–12 years, increasing to 50% for cats and 68% for dogs aged over 15 years.^6,33-35^ Companion animals unaffected by cognitive dysfunction syndrome remain cognitively intact, ageing normally, a feature found in humans.^6,33^ Comorbidities in ageing animals, including degenerative joint disease, hyperthyroidism and diabetes, can cause altered behaviours confounding cognitive dysfunction syndrome diagnosis. Therefore, diagnosing cognitive dysfunction syndrome requires observing animals’ behaviours during daily activities rather than the interviews and cognitive assessments used in humans.^34,36,37^

In humans, behavioural and psychological symptoms of dementia (BPSD) define the cognitive, psychological and clinical signs that are severe enough to negatively impact or prevent entirely the daily routine of dementia sufferers.^38^ BPSD commonly include memory loss, dysregulation of emotion, thought and personality and motor function deterioration,^4,39^ with specific symptoms of delusion, hallucination, aggression, depression or anxiety, extreme elation and abnormal sleep and wake cycles, amongst others.^38^

### Dogs

Ageing dogs with cognitive dysfunction syndrome show disorientation, impaired social interaction, altered house training and changes in sleep–wake cycles when cognitive function is assessed in a wide range of breeds of varying ages.^35,40^ Dogs aged 11–16 years showed progressive cognitive impairment over a 6–18-month period, with greater progression in dogs with at least one impairment at the onset of the study than those without any.^40^ This is similar to humans in that BPSD occur in ∼90% of dementia patients, with ∼50% presenting with at least four BPSD simultaneously.^38,39,41^ The mean age of onset of impairment in dogs is 11 years, ∼75% of the mean 15-year lifespan, and aligns with the mean age of human dementia diagnosis between 70 and 80 years.^28,38^

In dogs, a variety of cognitive tests investigate spatial, discrimination and reversal learning to assess spatial awareness, object and social recognition and memory through task learning and ‘unlearning’.^6,10^ Ageing dogs display human-like age-related social changes, including social withdrawal (e.g. lack of interest in others or available toys) or lack of self-recognition (e.g. increased interaction with own reflection).^10,42^ Studying social interaction in companion animals is invaluable as this cannot be assessed to the same extent in laboratory rodents due to their simplified brain anatomy and lack of training and communication.^6^

**Figure 2:**
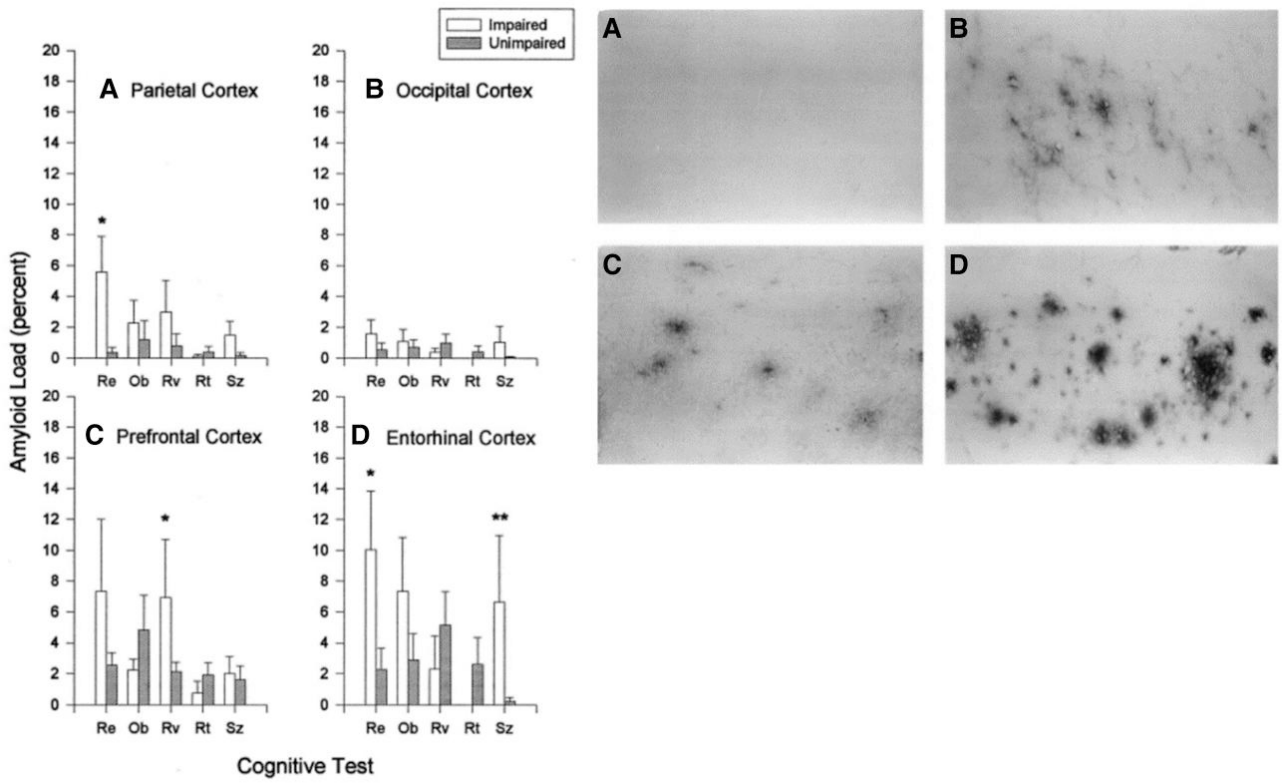
**The association between cerebral Aβ plaques and cognitive dysfunction in aged dogs.** Left panel: distribution of amyloid in the brains of impaired and unimpaired dogs and the relationship with cognitive tasks; note significant differences in the prefrontal and entorhinal cortices (*P < 0.05; **P < 0.075). Re, reward-approach learning; Ob, object-approach learning; Rv, reversal learning; Rt, retention testing; Sz, size-discrimination learning. Right panel: photomicrographs of Aβ deposition, using immunoreactivity, in the prefrontal cortex of four beagles: (**A**) young, (**B**) unimpaired middle-aged, (**C**) unimpaired aged, (**D**) impaired aged.^10^

Dogs show a stronger association between pathological lesions and behavioural changes than cats, especially cerebral Aβ deposition, hyperphosphorylated tau and neuronal loss.^10,33-35,43,44^ Similar to humans with Alzheimer's disease, ageing impaired dogs often have more Aβ in the prefrontal and the entorhinal cortices (Fig. 2), and dogs aged 2–13 years have a significant correlation between Aβ load in the prefrontal lobe with decreased abilities in object and visual discrimination learning, whereas Aβ load in the entorhinal cortex correlates more with size discrimination and object-approach learning.^10^

Additionally, humans develop cerebral amyloid angiopathy, defined as an impaired or inability to clear Aβ causing an accumulation of Aβ in the cerebral arteries, which causes vascular cognitive impairment most commonly in the form of vascular dementia.^45-47^ Similarly, dogs frequently develop Aβ deposition in the cerebral blood vessels causing cerebral amyloid angiopathy^6^ supporting the significance of Aβ pathology causing the clinical signs seen in ageing dogs.

Yu *et al.*^37^ evaluated the brains of 10 dogs with cognitive dysfunction after ruling out comorbidities and found a significant age-dependent increase in Aβ and hyperphosphorylated tau in impaired dogs’ cerebral cortices and hippocampi compared to healthy controls. However, expression of p-tau Ser396 and accumulation of ubiquitin were significantly increased in the parietal cortex and dorsal part of the hippocampus of the brain of aged dogs compared to humans with Alzheimer’s disease, Braak Stage V, potentially due to faster accumulation in dog brains than human brains in the earlier stages of cognitive dysfunction. Interestingly, no reports of fully formed mature NFTs in aged, cognitively impaired canine brains exist.^6,37,48^ Further investigation of more dogs is necessary, but several possible explanations exist for these discrepancies. One suggestion is that a dog's lifespan is too short to allow mature NFTs to develop; another suggests limited phosphorylation sites exist in dog tau compared to human tau, therefore preventing NFT development.^37,49^ Another is that all the studies that failed to find mature NFTs in dogs used antibody AT8, which recognizes Ser202, a Thr205 epitope of hyperphosphorylated tau,^6,37,48^ whereas one that found early NFTs used an antibody targeting the earlier forming Ser396 epitope.^50^ Furthermore, most research of dementia-like pathology in the brains of dogs has been performed in laboratory beagles, but a study examining other breeds showed breed-related differences in Aβ accumulation, particularly with respect to age of onset.^51^

Cognitively impaired dogs develop additional human-like pathology including neuronal loss and brain atrophy, ventricular enlargement, reduced neurogenesis and increased lipofuscin accumulation.^6,35,42,48^ Yu *et al.*^37^ reported significant neuronal loss in the cornu ammonis 1 (CA1) hippocampal region in cognitively impaired dogs, with less than half the average number of neurons present in healthy controls, and Siwak-Tapp *et al*.^42^ estimated up to 30% neuronal loss in the hippocampus and 90–95% neurogenesis decline in aged beagles. Studies found macroscopic brain atrophy with functional topography beginning in the prefrontal and mesial temporal cortices as early as 8 years old, 53% of mean dog lifespan, followed by hippocampal atrophy after ∼75% of the mean lifespan, at 11 years old.^29,42,52^ These regions align with the mesial temporal lobe and the entorhinal cortex of the human brain, which are susceptible to pronounced atrophy in patients with Alzheimer's disease^53^ and suggest, similar to ageing human brains, specific regions of the ageing dog brain are more vulnerable to neurodegeneration.^48,49^ However, another study found no macroscopic atrophy.^37^

It should be noted that laboratory beagles have been used in the pharmaceutical industry for testing potential therapeutic interventions for Alzheimer's disease.

### Cats

Less evidence exists for direct clinicopathological correlation between age-related pathology and cognitive dysfunction in cats compared to dogs.^34,48^ However, cognitive dysfunction syndrome in cats is widely acknowledged, and the associated behavioural and psychological changes are described by V-I-S-H-D-A-A-L (*v*ocalization increase; *i*nteraction with owners or other pets; *s*leep–wake cycle changes; *h*ouse soiling; *d*isorientation; *a*ctivity changes; *a*nxiety; and *l*earning and memory),^6,54,55^ which are comparable to the BPSD in humans,^38^ although cats in the most recent study did not have a diagnosis of cognitive dysfunction.^52^

Recent discussions have suggested abnormal behaviours in ageing cats include aberrant vocalizations.^55,56^ Although these vocalizations are challenging to determine, they are a likely indicator of cognitive dysfunction syndrome in the absence of other diseases. Černá *et al.*^57^ investigated unusual vocalization in 37 aged cats with cognitive dysfunction syndrome, upon exclusion of other comorbidities, and owners suggested disorientation, attention-seeking, hunger or pain causing this behaviour in some cases. More extensive studies, including one investigating 883 cats older than 11 years, found excessive vocalization in ∼60%.^58^ Aberrant vocalizations in ageing cats likely align with abnormal and inappropriate outbursts comparable to agitation or aggression displayed by humans with dementia.^38^ Further investigation into the pathological features of cats with aberrant vocalization is necessary to compare this behaviour with humans.

Cognitive tests, similar to those used for dogs,^6,10^ advance the interpretations of owner observations. These tests show an age-related decline in cognitive abilities, the progression of which is estimated to begin around 10 years of age, or 60–70% of average life expectancy of the companion cat, which is considered geriatric by 15 years,^55,56^ aligning with the average age at which dementia symptoms occur in humans.^38^

Examination of ageing cat brains shows Aβ plaques in the neuropil and the cerebral blood vessels,^34,48^ although some suggest cats only develop diffuse deposits of Aβ representative of early or pre-plaque formation.^30,33,59,60^ In humans, dementia-associated plaques consist predominantly of Aβ composed of peptides of 40 or 42 amino acid residues (Aβ40 and Aβ42, respectively).^6,61^ Aβ42, due to its larger size, lower solubility and increased fibrillization, is more abundant than Aβ40, but both are present.^62^ The plaques found in ageing cat brains are composed of Aβ42, not Aβ40, whereas the vascular amyloid angiopathy is composed of both Aβ40 and Aβ42,^33,59^ identical to the vascular amyloid composition in humans with dementia.^62^ The relationship between amyloid pathology and cognitive dysfunction in ageing cats is debated as there is no clear evidence of correlation between the amount and distribution of Aβ in the brain with the severity of cognitive dysfunction.^30,33,48^

**Figure 3:**
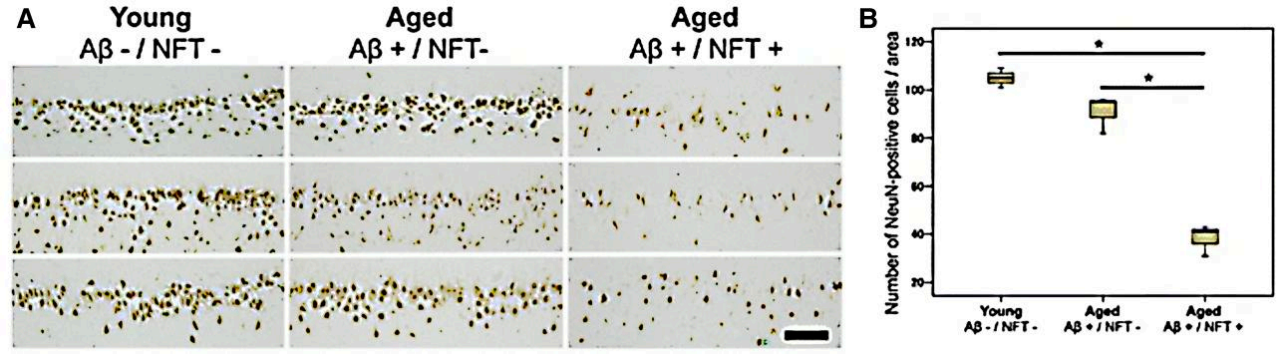
**Neuronal load in the hippocampal regions of young and aged cats.** (**A**) Immunohistochemistry detecting NeuN (a protein used to detect mature neurons) load in one young (Aβ−/NFT−; *n* = 3; mean age = 3.7 years) and two aged groups (Aβ+/NFT−; *n* = 3; mean age = 18 years and Aβ+/NFT+; *n* = 3; mean age = 17.6 years) with or without Aβ and NFT pathology (scale bar = 100 µm). (**B**) Quantification of the number of NeuN-positive cells in the hippocampal regions of the cats.^64^ *P < 0.001. Aβ, amyloid-beta; NFTs, neurofibrillary tangles.

Ageing cats with cognitive dysfunction syndrome rarely display fully formed NFTs,^30,33,34^ but they do have five of the six normal hyperphosphorylated tau isoforms identical to humans.^26,34^ Hyperphosphorylated tau is neurotoxic in humans, causing a loss of the protein's normal function and promoting aggregation.^63^ As such, the hyperphosphorylated tau in ageing cat brains suggests the possibility of pre-tangles that could develop later into pathological NFTs, but the shorter lifespans of cats compared with humans may reduce this phenomenon.^34,49,55^ However, one study reported an association between the presence of pre-tangles and cognitive dysfunction syndrome,^60^ whilst another study reported fully formed NFTs present in the hippocampus of a few aged cats.^64^ Comparisons of neuronal loss in cats with and without Aβ or NFTs found a significant correlation between neuronal loss and NFT formation as aged groups without NFT pathology showed similar neuron load to young cats with no Aβ accumulation or NFT (Fig. 3).^64^ This suggests NFT pathology is more closely associated with neuronal loss than Aβ accumulation in cats and possibly a more accurate biomarker for cognitive decline in ageing cats.^55^ Chambers *et al.*^64^ measured neuronal loss in cats post-mortem using immunohistochemistry and found that Aβ accumulation precedes tau accumulation and neuronal loss in ageing cats.

Although domestic cats and dogs display features of human dementia, further investigation should focus on the differences such as the absence of NFT in aged, cognitively impaired dogs yet its presence in some aged cognitively impaired cats. As hyperphosphorylated tau exists in these animals and is a hallmark pathological feature of Alzheimer's disease, understanding the progression from hyperphosphorylated tau to NFT formation and its implications on cognitive decline is critical to determine the full potential of these animals as research models for this disease.^48^ Chambers *et al.*^64^ suggest that cats develop NFT, but aged dogs do not, possibly due to the involvement of Aβ in furthering tau pathology, and vice versa. As such, one hypothesis highlights the disparity in amyloid peptide amino acid sequences between cats and dogs (Table 1).^64^ Human and dog amyloid peptide amino acid sequences are 100% homologous.^29^ However, cats differ at the seventh residue, and it has been suggested this promotes more extensive Aβ accumulation.^64^ It is likely that this greater amyloid load triggers increased tau pathology, resulting in the NFT formation found in cats, but not in dogs.^64^

## Farm animals

Farm animals are increasingly studied as potential models for human dementia due to their similar size and complexity with humans compared to rodents (Fig. 1).^25^

### Pigs

The pig brain closely resembles the human brain,^13,65,66^ being gyrencephalic with similar development in late gestation and early postnatal life.^13,66^ Furthermore, the prefrontal cortex, a key region in human neurodegeneration, is ∼10% of the pig brain, similar to humans.^13^

Pigs live in small groups composed of a female and her offspring, and adult males are solitary, with interaction only occurring during the mating season. Pigs recognize familiar people and objects, but as the porcine visual system is less developed than non-human primates and humans, much of this recognition relies on auditory and olfactory senses.^13,67^ Additionally, like humans, pigs are able to relate cognition with emotion, displaying positive or negative reactions to situations in adapting to changing environments. For example, pigs recognize danger and associate negative emotions such as fear or anxiety with them, in the form of ‘freezing’, ‘standing alert’, defaecation and urination, and vocalizations.^13^ These complex cognitive abilities make pigs potentially better models for studying human dementia as they may be more permissive to cognitive testing.^13,65^

Pig behaviour, spatial discrimination and cognitive ability have been investigated using mazes.^13,66^ However, studying cognitive decline in pigs has limitations, including size, cost, space requirements and specialized housing,^65^ which are all significantly less in rodent studies. However, the larger brains and the existence of tau isoforms in pigs that are identical to human isoforms raise the potential of pigs as candidates for studying human dementia.^65^

Similar to the induction of the amyloid precursor protein (APP) gene to trigger the production of Aβ in the transgenic mouse model,^68^ Kragh *et al.*^69^ developed the first transgenic pig model using a mutation associated with early onset of Alzheimer's disease in humans to induce expression of human APP, and this induced increased expression of Aβ42 and accumulation,^65^ the primary component of human amyloid plaques.^61^ Although tau pathology and NFT formation have not been identified in ageing pigs, they do express four of the six isoforms found in humans,^26^ whereas wild-type rodents have no human tau isoforms.^65^ Compared with other animals, interest in pigs for cognitive research is relatively new^13^; therefore, more extensive studies on ageing pig pathology and behaviour are required to more accurately correlate pathology with cognitive decline.

### Equids

Horses’ cognition is considered high due to their use in sport, work and human companionship,^70^ and so their relationship with humans is similar to dogs and cats rather than farm animals. This provides an opportunity to study the social interaction components of cognitive function and subsequent dysfunction.^70^ Evidence suggests horses have complex abilities of discrimination and memory. Lampe and Andre^71^ reported that horses could use visual, olfactory and auditory cues to recognize individual humans and associate positive or negative emotions with those individuals, suggesting a relationship between affect and cognition, as found in pigs.^13^ Although horses have not been systematically exposed to as many cognitive tests as cats or dogs, observations of memory loss indicators (e.g. confusion, disorientation and altered social behaviour) in aged horses have been reported.^6^

Two studies have investigated pathological lesions in ageing horses: one that studied 60 horses aged 7–23 years^12^ and the other that studied 100 horses aged 2–25 years.^11^ Both studies reported age-related increases in neuronal lipofuscin accumulation and age-related vascular degeneration and neuronal vacuolation.^11,12^ Lipofuscin pigment accumulation in neurons is associated with ageing in humans, commencing at age 9 and increasing with age,^72-74^ and abnormal aggregation and accumulation is present in many neurodegenerative diseases. While neither study in horses found amyloid plaques, possibly due to the use of Congo Red stain only rather than immunohistochemistry, or NFTs, Capucchio *et al.*^12^ reported tau-positive neurons and methenamine-positive diffuse plaques potentially indicating pre-NFT and pre-amyloid plaques, respectively, but only one of the horses displayed both. Along with horses, dogs and cattle have also shown specific aberrant lipofuscin accumulation in line with other neuropathology and signs of cognitive dysfunction,^6,75^ and this may be a potential biomarker for dementia-like pathology in non-human animals.

Although horses have been at the forefront of studies in equids, Malbon *et al.*^15^ conducted a post-mortem study on 13 donkeys aged between 33 and 44 years, with two younger controls (aged 8 and 9), to assess donkeys as a potential model for human neurodegeneration. Aβ plaques were identified in nine and NFT-like structures in seven of the aged donkeys, whilst neither of the younger donkeys displayed either pathology. Furthermore, because the oldest donkey in the sample (aged 44) displayed neither pathology, it is suggested that these lesions may not be part of the normal ageing process in donkeys.^15^ Additionally, this study highlighted the potentially significant association between Type II diabetes mellitus, obesity and Alzheimer's disease found in humans and the role of insulin dysregulation in these processes.^76^ Interestingly, equine metabolic syndrome is common in several equid species and is the most common endocrine disorder in donkeys.^15,77^ In equine metabolic syndrome, obesity causes reduced insulin clearance and adipokine (e.g. leptin and adiponectin) dysregulation, which can lead to hyperinsulinaemia.^15,77^ In humans, leptin and adiponectin are closely associated with Alzheimer's disease development^78^ and therefore present a promising opportunity for research in donkeys into their roles.

As the study by Malbon *et al.*^15^ remains the only published study that explored Alzheimer's disease-like pathology in donkeys and used brains from animals already dead, behavioural assessments were not obtained prior to death. Therefore, further investigation is required to fully understand how the pathology identified relates to human neurodegeneration.

### Cattle and sheep

**Figure 4:**
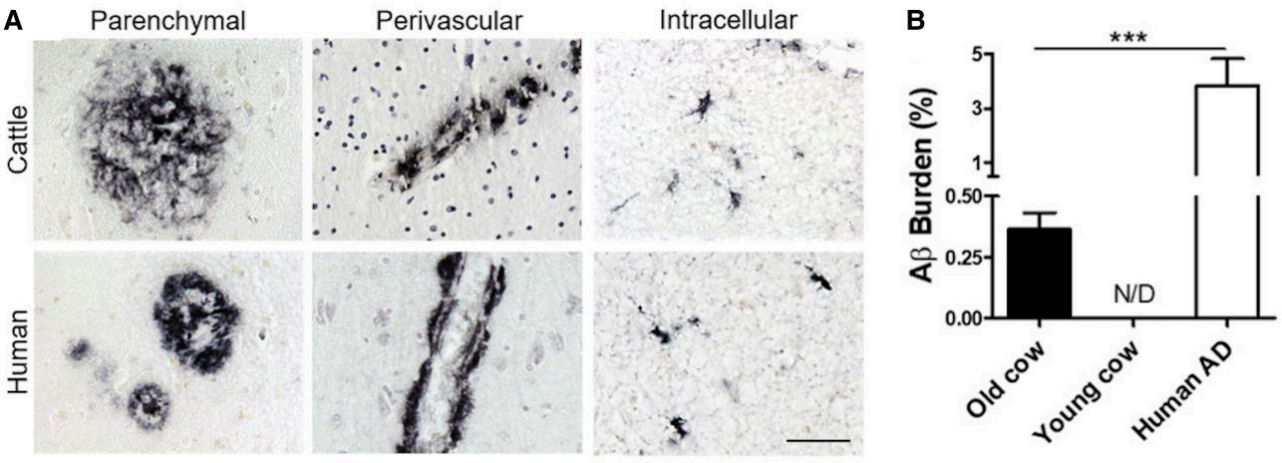
**Comparison of amyloid load in cattle versus humans.** (**A**) Anti-Aβ antibody labelling detecting parenchymal, perivascular and intracellular amyloid deposits in the temporal regions of aged cattle and aged human brains (scale bar = 50 µm). (**B**) Percentage of the brain occupied by Aβ plaques in the brains of old cattle (*n* = 63; age 13–23 years; Aβ burden 0.37 ± 0.07%) in comparison to healthy young cattle group (*n* = 10; age 10 months; Aβ burden not detected) and a human Alzheimer's disease group (Aβ burden 3.82 ± 1.0%). N/D denotes no Aβ aggregation detected.^14^

Like many other animals investigated, the Aβ sequence in cattle is identical to humans.^14^ A study using an anti-human Aβ antibody labelled human-like mature Aβ plaques in the cerebral cortices and hippocampi of 14 cattle over the age of 13 years (*n* = 63, age 13–23 years), and labelling was also present in the perivascular spaces, indicating cerebral amyloid angiopathy-like pathology (Fig. 4).^14^ All negative control cattle (*n* = 10, 10 months old) were devoid of labelling. The Aβ burden was much lower in aged cattle than humans with Alzheimer's disease, but the accumulation in cattle was age dependent (Fig. 4). However, another study in cattle (*n* = 102, age range 0–240 months) found intraneuronal and extra-neuronal deposits increased with age but also failed to find any Aβ plaques and suggested this may be due to a lack of β-sheet secondary protein structures in bovine Aβ.^79^

Sheep have been examined for suitability for genetic manipulation to produce a large animal model of Alzheimer’s disease due to the amino acid sequences of ovine and human Aβ40 and Aβ42 being identical, that of APP being highly conserved and the total levels of tau protein in cerebrospinal fluid being comparable.^80^ Furthermore, using anti-Aβ and phosphorylation-specific (AT8 and S396) anti-tau antibodies in the hippocampal and entorhinal regions of 30 sheep brains (estimated ages 1–2 years, 2–3 years and >5 years), Davies *et al.*^81^ reported both intracellular and vascular Aβ in sheep ≥2 years old, phosphorylated tau increasing with age and pre-NFT were present in sheep estimated to be >5 years old. Sheep express four isoforms identical to human tau, especially the 3R and 4R human isoforms, and in their pilot study, Davies *et al.*^81^ reported mature NFT formation in a 21-year-old sheep.

Although farm animals have the potential for developing spontaneous dementia-like pathology and cognitive decline, clinical investigation is more complicated than in laboratory or companion animals.^13^ The studies on horses,^11,12^ donkeys,^15^ cows^14^ and sheep^81^ were conducted on brains collected post-mortem with no ante-mortem assessment of cognitive decline undertaken. Farm animals are challenging to train for behavioural observations, making cognitive assessment difficult, but not impossible. However, given the social interactions between some equids and humans,^82^ further research on cognitive abilities in horses and donkeys may identify clinicopathological correlations. Also, as sheep show spontaneous development of Aβ deposition, pre-NFT and potentially mature NFT formation in old age^81^ like humans, therefore studying their behaviour may be beneficial too. It is worth noting that some transgenic pig^83^ and sheep^84^ models have been developed for neurodegenerative diseases other than Alzheimer's disease such as Huntington's disease.

## Marine mammals

Marine mammals have been subjected to the least amount of neurodegenerative research due to the difficulty in studying the progression of neurodegeneration and cognitive decline during the animals’ lifetimes. Thus, much of the research has focused on brains recovered after death due to stranding. Nonetheless, evidence for dementia-like pathology has been observed in several studies of odontocetes (toothed whales and dolphins)^18,85^ and pinnipeds (sea lions, seals and walruses),^16^ raising their importance as potential natural spontaneous models of dementia.^29^

### Odontocetes

**Figure 5:**
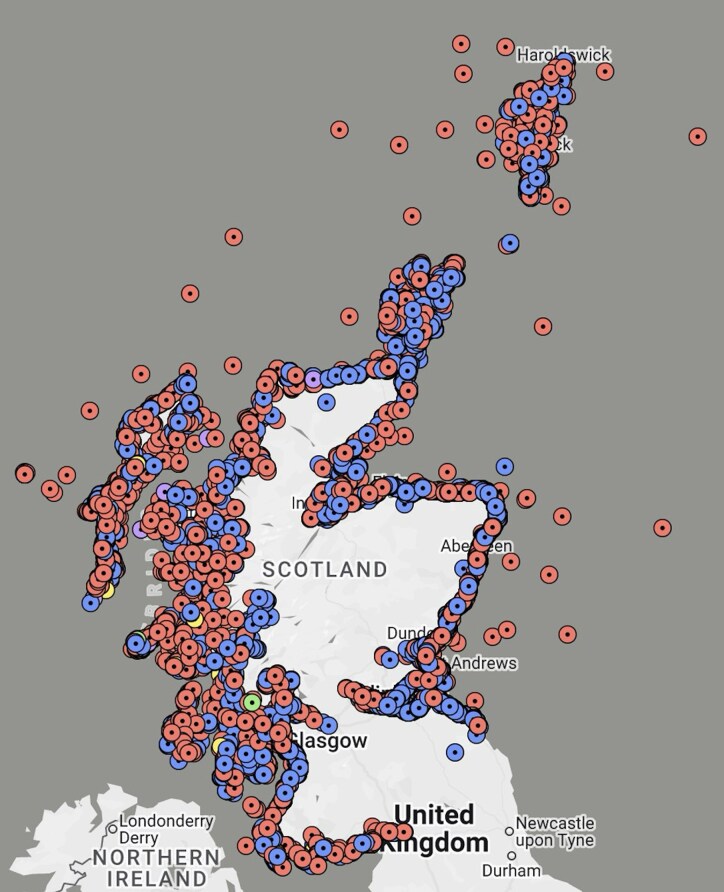
**Cetacean, pinniped, seas turtle and basking shark strandings in 2021 in Scottish coastal waters.** A map of 17 species of cetacean (red), pinniped (blue), turtle (yellow), basking shark (green) and other shark species (purple) reported stranded along the coast of Scotland in 2021 (*Taken from SMASS, 2021—Map of Strandings*).^87^

Odontocetes have grown in interest in cognitive dysfunction research for various reasons including comparable lifespans to humans, identical Aβ amino acid sequence to human Aβ in several odontocete species^29,85^ and the cooperative living practices of numerous species.^18^ Of particular interest is their epimeletic, or caregiving, behaviour towards injured or diseased individuals such as carrying an individual, keeping it afloat or protecting it from further danger that have all been observed more frequently in odontocetes than in other marine mammals.^18,86^ This behaviour is important in the study of neuropathology as the disease can progress further, allowing more advanced lesions to develop compared with solitary individuals that are less likely to survive to more advanced stages of the disease.^18^ Furthermore, epimeletic behaviour aligns more with human behaviour, allowing comparative evaluation of the social interaction components of cognition. The Scottish Marine Animal Stranding Scheme (SMASS)^87^ investigates stranded cetaceans, pinnipeds, marine turtles and sharks along the Scottish coast (Fig. 5) and was a source of material for a comprehensive study.^18^

A recent study by Vacher *et al.*^18^ investigated brains of 22 individuals from five different odontocete species that had stranded in Scotland. Using evidence of old age, such as worn or lost teeth, relative increases in cerebral white matter or life history-related photo identification, 18 of the 22 dolphins were designated as aged, including Risso's dolphins (*Grampus griseus*), long-finned pilot whales (*Globicephala melas*), white-beaked dolphins (*Lagenorhynchus albirostris*), harbour porpoises (*Phocoena phocoena*) and a bottlenose dolphin (*Tursiops truncatus*), and four were designated as young adults or subadults. All 18 aged odontocetes showed intracellular Aβ accumulation, especially in the limbic and paralimbic lobes that, in the odontocete brain, overlay the basal ganglia and the thalamus, respectively.^18,88,89^ Additionally, in the oldest animals, significant amounts of both intra- and extracellular deposits of Aβ were present in the brain regions corresponding to those in humans with Alzheimer's disease.^18^ Of the 18 aged odontocetes, three individuals (one *L. albirostris,* one *G. melas* and one *T. truncatus*) showed Aβ plaques, probable cerebral amyloid angiopathy (vascular Aβ accumulation) and hyperphosphorylated tau. This suggested a pre-NFT biomarker, and further investigation of these aged odontocetes showed both Aβ and tau aggregations in additional brain regions that were not observed in the four young adults or subadults, suggesting spontaneous, age-related protein aggregation and progression.^18^

Comparing odontocete brains with human and terrestrial animal brains is difficult as much of the odontocete brain has structurally adapted to an aquatic environment.^18,88,89^ However, the Aβ plaques found in the odontocetes’ brains by Vacher *et al.*^18^ were predominantly in the rostrolateral cortices, called the orbital lobe in odontocetes. The orbital lobe contains the limbic and paralimbic lobes and is considered homologous with the human frontal lobe.^18,89^ This aligns with the initial accumulation of amyloid plaques in the frontal, temporal and occipital lobes of human brains in Alzheimer's disease.^18,90^ Despite the similar overall size and complexity of the odontocete brain and the inclusion of all the human hippocampal regions (dentate gyrus, subiculum, hippocampus and entorhinal cortex) in the odontocete hippocampus, the latter is much smaller than in humans, estimated at only 10% of the size.^88,89^ These disparities in regional size raise curiosity about functional localization in odontocete brains. A reduction in the size of the hippocampus suggests a smaller reliance on this structure, with other more developed structures necessary for aquatic survival. Oelschläger^88^ suggested the reduced hippocampal size may correspond to the reduced olfactory system in cetaceans. Alternatively, as the hippocampus is associated with movement and spatial memory, a reduced hippocampus may be less vulnerable to injury, thereby reducing behaviours that may cause stranding.^18,88^ Unfortunately, in the archived brain samples used by Vacher *et al*.,^18^ the hippocampi were not sampled.

Evidence of Alzheimer's disease-like pathology, amyloid plaques and NFT, in odontocetes is insufficient to diagnose Alzheimer's disease as this requires identification of significant clinical cognitive dysfunction. However, studying odontocete behaviour is challenging as samples from free-ranging animals are only available after stranding. Although there are no definitive reports of cognitive decline in ageing odontocetes, the animals studied by Vacher *et al*.^18^ and others reported by SMASS^87^ (Fig. 5) all stranded. Live-strandings could result from pathology-associated disorientation, aligning with the first signs of dementia in humans and commonly presenting as confusion and poor sense of direction.^18,91^ The ‘sick-leader hypothesis’ has been proposed as a possible cause of mass-strandings in marine mammals, whereby an impaired leader erroneously leads a group into shallow waters.^18,91^ These conclusions, however, are purely speculative.

To investigate this hypothesis, further studies must compare Alzheimer's disease-like pathology with behavioural disparities, and this may be possible in captive odontocetes. Stylianaki *et al*.^92^ investigated Alzheimer's disease-like pathology in a 40-year-old captive bottlenose dolphin (*T. truncatus*) finding parenchymal (in the equivalent of the human frontal, temporal and parietal lobes) and vascular Aβ plaques. No mature NFTs were reported, but this study showed apolipoprotein E (APOE) immunoreactivity, similar to human Alzheimer's disease, suggesting a human-like involvement of the APOE gene in Aβ plaque formation.^92^ This dolphin showed no signs of cognitive dysfunction but was found with undigested fish in the stomach (usually indicative of sudden death as there is rapid transit of ingesta in cetaceans) and evidence of drowning, which may indicate some form of memory loss. Again, these conclusions are highly speculative and require further investigation.

Another suggestion for the observed neuropathology in odontocetes relates to their life histories. Some odontocetes are known to be deep-diving, frequently reaching depths of 1000 and even 3000 m, which a Cuvier's beaked whale (*Ziphius cavirostris*) surpassed for over 2 h.^85^ Diving to such depths repeatedly increases the risk of significant episodes of cerebral hypoxia, despite adaptation to such conditions, and hypoxia increases the risk of neurodegenerative pathology.^85^ Neuroglobin, a haem protein expressed within neurons with a higher affinity for oxygen than haemoglobin, enables oxygen to move from the vascular compartment to the neural tissue, providing oxygen stores to afford some protection under hypoxic conditions.^85^ However, diving mammals have the same amount of neuroglobin as non-diving mammals, so this mechanism does not negate the risk of hypoxia.^85^ Similar to ageing, cerebral hypoxia can lead to hypometabolism, which is known to trigger APP overexpression and prevent homeostatic clearance of Aβ aggregates in humans with dementia and can cause inflammation, oxidative stress and neuronal loss.^93^ These processes may be implicated in the neuropathologies reported in odontocete species.

### Pinnipeds

**Figure 6:**
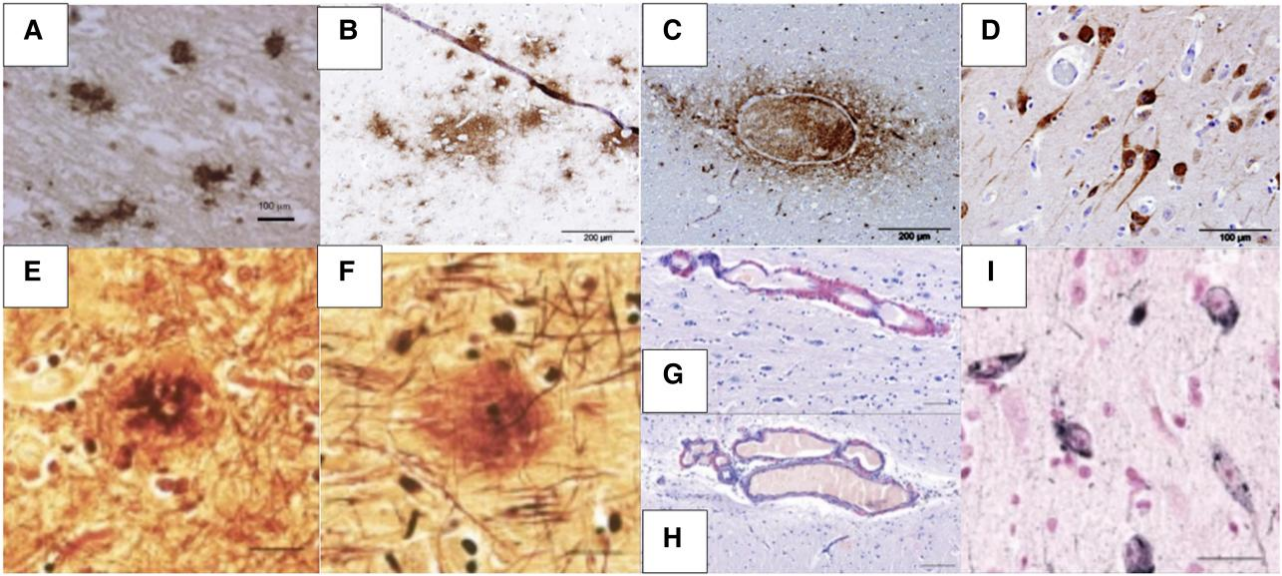
**Neuropathology in aged cetaceans and pinnipeds.** Amyloid plaques (brown pigment) in (**A**) a striped dolphin (*Stenella coeruleoalba*)^29^ (scale bar = 100 μm); (**B**) diffuse plaques in an aged bottlenose dolphin (*T. truncatus*)^18^ (scale bar = 200 μm); (**C**) amyloid plaque deposits around blood vessels indicating cerebral amyloid angiopathy in a white-beaked dolphin (*L. albirostris*)^18^ (scale bar = 200 μm); (**D**) axons and dendrites containing hyperphosphorylated tau (brown pigment) in an aged long-finned pilot whale (*G. melas*)^18^ (scale bar = 100 μm); (**E–I**) pathology in a 32-year-old Australian sea lion (*Neophoca cinerea*) showing (**E**) neuritic plaques (dark red pigment) with a dense core^16^ (scale bar = 20 μm); (**F**) diffuse plaques without a distinct core^16^ (scale bar = 20 μm); (**G**) blood vessels in the meninges with amyloid aggregation (red pigment)^16^ (scale bar = 50 μm); (**H**) blood vessels in the cerebral cortex with amyloid aggregation (red pigment)^16^ (scale bar = 100 μm); and (**I**) NFTs (black pigment) in the neurites of the cerebral cortex (scale bar = 50 μm).^16^

Although odontocetes have been more extensively investigated, several studies have assessed dementia-like pathology in aged pinnipeds, including seals (Phocidae), sea lions (Otariidae) and walruses (Odobenidae), which have also an identical Aβ peptide sequence to humans (Table 1). Takahashi *et al*.^16^ investigated the brains of 10 pinnipeds (four seals, five sea lions and one walrus) aged 0–35 years, and amyloid plaque pathology was present in eight of them (aged 27–35) in various combinations of the frontal, parietal, temporal and occipital lobes of the cerebral cortex, but none in the white matter, brain stem or cerebellum. Plaques were also present in the blood vessels, indicating cerebral amyloid angiopathy-like pathology (Fig. 6). Additionally, both neuritic plaques, with a distinct compact core, and diffuse plaques, devoid of a distinct core, were present, and both had a higher composition of Aβ42 than of Aβ40,^16^ aligning with Alzheimer's disease in humans.^61^ The depositions were considered to be age dependent as none were found in the two infant or juvenile pinnipeds examined, whereas all eight aged pinnipeds had amyloid plaques. Takaichi *et al*.^16^ also found an increased number and size of astrocytes and microglia with enlarged pre-synapses around amyloid deposits, a feature found commonly around amyloid plaques in humans suggestive of increased inflammation.^94^ Pinnipeds express five of the six human tau isoforms (1N3R, 2N3R, 0N4R, 1N4R and 2N4R), and significant NFT formation with fibrillar aggregation was reported in these pinnipeds.^16^

Pinnipeds have also shown human-like vascular Aβ deposits colocalized with APOE in the arterial and capillary walls.^16^ The eldest sea lions (aged 27–32 years) showed more severe cerebral amyloid angiopathy pathology compared to the younger pinnipeds and followed a similar regional progression in the frontal, temporal, parietal and occipital lobes^16^ as humans with dementia.^46^ Although the findings of this report seem promising, it was the first study to report spontaneous Alzheimer's disease-like pathology in aged pinnipeds and further investigation is required.

## Discussion

With an ageing population and an increase in dementia cases worldwide, the search for a cure or even a treatment to slow the progression of cognitive decline is critical. Animal models are vital in scientific research to study and understand many human diseases and their pathogeneses. Although rodents have been used extensively for cognitive research, due to ease of use, accessibility, cost and ethical and legal restrictions, recent studies have suggested that larger animals with brain anatomy and lifespans more similar to humans should be considered. Through analysis of the published literature, this review has identified animal species that spontaneously develop age-related neurodegeneration. Youssef *et al.*^6^ published a review of the evidence for dementia-like pathology in cats, dogs, horses and primates, discussing some of the behaviours likely to be associated with this pathology. However, this review provides a deeper understanding of the pathological features and several additional animal species and highlights the clinicopathological correlations in all these animal species.

Ambiguity remains with respect to the pathological lesion(s) most representative of neurodegeneration. Several studies propose it is the sequence of onset and progression of amyloid and tau pathologies,^95-97^ whilst others suggest that neuronal loss is the most important pathological feature.^41,98-100^ Furthermore, loss of synapses is considered to be the strongest correlate of cognitive decline in Alzheimer's disease.^101-104^ As the primary proteinopathies in Alzheimer's disease, amyloid and tau accumulations represented by Aβ plaques and NFTs, respectively, have been the primary focus of studies of pathology relating to neurodegeneration in ageing animals (Table 3). However, to fully assess these non-laboratory animal species as potential models of wider human dementia, it is essential that the other proteinopathies present in humans, such as α-synuclein, Lewy bodies and trans-activation response element DNA binding protein 43 (TDP-43), are also investigated.^3,6,7^ Therefore, the significant proteinopathy and vascular pathologies present in several species of ageing animals represent promising subjects for further investigation (Table 3). Ultimately, to identify the animal(s) that model human diseases most accurately, all aspects of Alzheimer's disease pathology must be thoroughly studied, which will include determining other diseases responsible for causing cognitive dysfunction in these various animal species.

**Table 3:** Summary of the common pathological features found in human patients with dementia that have been reported in animal species suggesting their potential as models for studying these specific aspects in human dementia

		Amyloid pathology	Tau pathology	Brain atrophy	Neuronal loss	Pigment accumulation	Vascular pathology
	Humans	Aβ plaques	NFT	+	+	+	Cerebral amyloid angiopathy
Companion animals	Dogs	Aβ plaques	Hyperphosphorylated tau	+	+	Lipofuscin accumulation	Cerebral amyloid angiopathy
	Cats	Aβ plaques	Pre-tangles/NFT	D.D.	+	D.D.	Cerebral amyloid angiopathy
	Horses	Pre-plaques	Hyperphosphorylated tau	D.D.	D.D.	Lipofuscin accumulation	Vascular degeneration
Farm animals	Pigs	Aβ42	−	D.D.	D.D.	D.D.	D.D.
	Donkeys	Aβ plaques	NFT-like structures	D.D.	D.D.	D.D.	D.D.
	Cattle	Aβ plaques	D.D.	D.D.	D.D.	D.D.	Cerebral amyloid angiopathy
	Sheep	Aβ plaques	Hyperphosphorylated tau/pre-tangles/NFT	D.D.	D.D.	D.D.	Vascular Aβ
Marine mammals	Odontocetes	Aβ plaques	Hyperphosphorylated tau	D.D.	D.D.	D.D.	Vascular Aβ
	Pinnipeds	Aβ plaques	NFT	D.D.	D.D.	D.D.	Vascular Aβ

D.D. denotes areas that are data deficient.

Studies of non-laboratory animal pathology have been limited in sample size, especially marine mammals due to their habitats limiting investigations of their brains to, primarily, those of stranded animals post-mortem.^16-18,85^ Furthermore, age is a critical factor in dementia, and the ages of the marine mammals available are usually estimated via subjective features that may lack accuracy.^18^ Impaired cognition is a critical component of human dementia diagnosis. Therefore, understanding impaired cognition and behaviour in animals is essential to identify the animal species that best models human dementia. As the conclusions drawn from many of these studies are based on extrapolations and speculations, larger sample sizes, ante-mortem assessments and full post-mortem investigations are required to validate any conclusions based on the cause of death, age at death and evidence of behavioural change and cognitive decline, and this must correlate with the types and extents of the pathology present.

However, there are several ethical and logistical limitations to studying behaviour in non-laboratory animals to the same extent possible in humans, resulting in dementia research in animals focusing primarily on post-mortem studies as behavioural observations were unavailable. Animal welfare concerns in several of the discussed candidate models pertain mainly to pain and suffering, general health and cognitive welfare. Although some animal research requires only observation of an animal's behaviour in their natural habitat, some can be invasive, harmful and/or painful to the subjects. The UK Animal Welfare Act 2006 holds owners legally accountable for ensuring the welfare of livestock and companion animals and preventing unnecessary pain and suffering.^105^ Additionally, the UK Parliament has recently dedicated discussion to the ethics and practicality of using animals in scientific and medical research, covered by the Animals (Scientific Procedures) Act 1986^106^ and its various amendments, and if legislation changes, some candidate animal species may become less available for scientific research. Moreover, 41 000 species are currently threatened by extinction,^107^ including many marine mammals, and this raises ethical considerations in the search for an animal model for better understanding human dementia, irrespective of how valuable such a model may be.

## Conclusion

Identifying and validating animal models of human diseases is complex and time-consuming. Human Alzheimer's disease and other dementias are, at present, devoid of any valid animal models representing of all the associated pathophysiological processes and behaviours of any individual disease, and also for assessing potential therapeutics. Although no single animal species develops all the characteristics of any human dementia, many develop proteinopathies, resulting in dementia-like pathology, and some have comparable and correlated cognitive dysfunction. Further and more comprehensive investigation of the pathological features and their correlations with clinical signs, especially cognitive dysfunction, in complex animals will identify the most appropriate model for studying this devastating, yet increasingly prevalent, group of diseases.

## Data Availability

Data sharing is not applicable to this article as no new data were created or analysed in this study.

## References

[1] Alzheimer’s Disease International . World Alzheimer Report 2015 The Global Impact of Dementia An analysis of prevalence, incidence, cost and trends . Accessed 23 January 2023. https://www.alzint.org/u/WorldAlzheimerReport2015.pdf

[2] Alzheimer’s Research UK. Prevalence and incidence of dementia . Accessed 21 January 2023. https://dementiastatistics.org/about-dementia/prevalence-and-incidence/

[3] Alzheimer’s Disease International. Dementia facts & figures . Accessed 03 April 2023. https://www.alzint.org/about/dementia-facts-figures/

[4] Dementia UK. What is dementia? . Accessed 21 January 2023. https://www.dementiauk.org/information-and-support/about-dementia/what-is-dementia/

[5] World Health Organization (WHO). ICD-11 for Mortality and Morbidity Statistics . Accessed 30 March 2023. https://icd.who.int/browse/2024-01/mms/en#213458094

[6] Youssef SA, Capucchio MT, Rofina JE, et al Pathology of the aging brain in domestic and laboratory animals, and animal models of human neurodegenerative diseases. Vet Pathol. 2016;53(2):327–348. 26869150 10.1177/0300985815623997

[7] Pendlebury ST, Rothwell PM. Prevalence, incidence, and factors associated with pre-stroke and post-stroke dementia: A systematic review and meta-analysis. Lancet Neurol. 2009;8(11):1006–1018. 19782001 10.1016/S1474-4422(09)70236-4

[8] Phillips NLH, Roth TL. Animal models and their contribution to our understanding of the relationship between environments, epigenetic modifications, and behavior. Genes (Basel). 2019;10(1):47. 30650619 10.3390/genes10010047 PMC6357183

[9] Levine MS, Lloyd RL, Fisher RS, et al Sensory, motor and cognitive alterations in aged cats. Neurobiol Aging. 1987;8(3):253–263. 3600956 10.1016/0197-4580(87)90010-8

[10] Head E, Callahan H, Muggenburg BA, Cotman CW, Milgram NW. Visual-discrimination learning ability and beta-amyloid accumulation in the dog. Neurobiol Aging. 1998;19(5):415–425. 9880044 10.1016/s0197-4580(98)00084-0

[11] Jahns H, Callanan JJ, McElroy MC, et al Age-related and non-age-related changes in 100 surveyed horse brains. Vet Pathol. 2006;43(5):740–750. 16966453 10.1354/vp.43-5-740

[12] Capucchio MT, Márquez M, Pregel P, et al Parenchymal and vascular lesions in ageing equine brains: Histological and immunohistochemical studies. J Comp Pathol. 2010;142(1):61–73. 19744668 10.1016/j.jcpa.2009.07.007

[13] Kornum BR, Knudsen GM. Cognitive testing of pigs (*Sus scrofa*) in translational biobehavioral research. Neurosci Biobehav Rev. 2011;35(3):437–451. 20553757 10.1016/j.neubiorev.2010.05.004

[14] Moreno-Gonzalez I, Edwards G, Morales R, et al Aged cattle brain displays Alzheimer’s disease-like pathology and promotes brain amyloidosis in a transgenic animal model. Front Aging Neurosci. 2022;13:815361. 35173603 10.3389/fnagi.2021.815361 PMC8841674

[15] Malbon AJ, Sordo L, Wilson LA, et al Alzheimer-like pathology in the parietal cortex and hippocampus of aged donkeys. Neurobiol Aging. 2022;113:7–14. 35278749 10.1016/j.neurobiolaging.2022.01.007

[16] Takaichi Y, Chambers JK, Takahashi K, et al Amyloid β and tau pathology in brains of aged pinniped species (sea lion, seal, and walrus). Acta Neuropathol Commun. 2021;9(1):10. 33413691 10.1186/s40478-020-01104-3 PMC7792306

[17] Takahashi K, Chambers JK, Takaichi Y, Uchida K. Different Aβ43 deposition patterns in the brains of aged dogs, sea lions, and cats. J Vet Med Sci. 2022;84(12):1563–1573. 36288928 10.1292/jvms.22-0386 PMC9791235

[18] Vacher MC, Durrant CS, Rose J, et al Alzheimer’s disease-like neuropathology in three species of oceanic dolphin. Eur J Neurosci. 2023;57(7):1161–1179. 36514861 10.1111/ejn.15900 PMC10947196

[19] Hooper L, Brosan FS. Primate cognition. Nat Educ Knowl. 2012;5:3.

[20] Hedges D, Farrer TJ, Bigler ED, Hopkins RO, editors. The brain at risk: Associations between disease and cognition. Springer; 2019.

[21] Dalley AF, Agur AMR, Moore KL. Moore’s clinically oriented anatomy. Wolters Kluwer; 2023.

[22] Henshaw JM . A tour of the senses. How your brain interprets the world. Johns Hopkins University Press; 2012.

[23] Didier ES, MacLean AG, Mohan M, et al Contributions of nonhuman primates to research on aging. Vet Pathol. 2016;53(2):277–290. 26869153 10.1177/0300985815622974 PMC5027759

[24] Jerison HJ . Evolution the brain and intelligence. Academic Press; 1973.

[25] Sorby-Adams AJ, Vink R, Turner RJ. Large animals as models of stroke and traumatic brain injury as translational tools. Am J Physiol Regul Integr Comp Physiol. 2018;315(2):R165–R190. 29537289 10.1152/ajpregu.00163.2017

[26] Janke C, Beck M, Stahl T, et al Phylogenetic diversity of the expression of the microtubule-associated protein tau: Implications for neurodegenerative disorders. Brain Res Mol Brain Res. 1999;68(1–2):119–128. 10320789 10.1016/s0169-328x(99)00079-0

[27] Podlisny MB, Tolan DR, Selkoe DJ. Homology of the amyloid beta protein precursor in monkey and human supports a primate model for beta amyloidosis in Alzheimer’s disease. Am J Pathol. 1991;138(6):1423–1435. 1905108 PMC1886384

[28] Götz J, Ittner LM. Animal models of Alzheimer’s disease and frontotemporal dementia. Nat Rev Neurosci. 2008;9(7):532–544. 18568014 10.1038/nrn2420

[29] Gunn-Moore D, Kaidanovich-Beilin O, Iradi MCG, et al Alzheimer’s disease in humans and other animals: A consequence of postreproductive life span and longevity rather than aging. Alzheimers Dement. 2018;14(2):195–204. 28972881 10.1016/j.jalz.2017.08.014

[30] Head E, Moffat K, Das P, et al Beta-amyloid deposition and tau phosphorylation in clinically characterized aged cats. Neurobiol Aging. 2005;26(5):749–763. 15708450 10.1016/j.neurobiolaging.2004.06.015

[31] Gunn-Moore D . Considering older cats. J Small Anim Pract. 2006;47(8):430–431. 16911109 10.1111/j.1748-5827.2006.00199.x

[32] Montoya M, Morrison JA, Arrignon F, et al Life expectancy tables for dogs and cats derived from clinical data. Front Vet Sci. 2023;10:1082102. 36896289 10.3389/fvets.2023.1082102 PMC9989186

[33] Gunn-Moore DA, McVee J, Bradshaw JM, et al Ageing changes in cat brains demonstrated by beta-amyloid and AT8-immunoreactive phosphorylated tau deposits. J Feline Med Surg. 2006;8(4):234–242. 16603401 10.1016/j.jfms.2006.01.003 PMC10822537

[34] Gunn-Moore D, Moffat K, Christie L-A, Head E. Cognitive dysfunction and the neurobiology of ageing cats. J Small Anim Pract. 2007;48(10):546–553. 17617164 10.1111/j.1748-5827.2007.00386.x

[35] Madari A, Farbakova J, Katina S, et al Assessment of severity and progression of canine cognitive dysfunction syndrome using the Canine Dementia Scale (CADES). Appl Anim Behav Sci. 2015;171(2):138–145.

[36] Gunn-Moore DA . Cognitive dysfunction in cats: Clinical assessment and management. Top Companion Anim Med. 2011;26(1):17–24. 21435622 10.1053/j.tcam.2011.01.005

[37] Yu CH, Song GS, Yhee JY, et al Histopathological and immunohistochemical comparison of the brain of human patients with Alzheimer’s disease and the brain of aged dogs with cognitive dysfunction. J Comp Pathol. 2011;145(1):45–58. 21256508 10.1016/j.jcpa.2010.11.004

[38] Schwertner E, Pereira JB, Xu H, et al Behavioral and psychological symptoms of dementia in different dementia disorders: A large-scale study of 10,000 individuals. J Alzheimers Dis. 2022;87(3):1307–1318. 35491774 10.3233/JAD-215198 PMC9198804

[39] Cerejeira J, Lagarto L, Mukaetova-Ladinska EB. Behavioral and psychological symptoms of dementia. Front Neurol. 2012;3:73. 22586419 10.3389/fneur.2012.00073 PMC3345875

[40] Bain MJ, Hart BL, Cliff KD, Ruehl WW. Predicting behavioural changes associated with age-related cognitive impairment in dogs. J Am Vet Med Assoc. 2001;218(11):1792–1795. 11394832 10.2460/javma.2001.218.1792

[41] Chiu MJ, Chen TF, Yip PK, Hua MS, Tang LY. Behavioral and psychologic symptoms in different types of dementia. J Formos Med Assoc. 2006;105(7):556–562. 16877235 10.1016/S0929-6646(09)60150-9

[42] Siwak-Tapp CT, Head E, Muggenburg BA, et al Neurogenesis decreases with age in the canine hippocampus and correlates with cognitive function. Neurobiol Learn Mem. 2007;88(2):249–259. 17587610 10.1016/j.nlm.2007.05.001 PMC2173881

[43] Cummings BJ, Head E, Afagh AJ, et al Beta-amyloid accumulation correlates with cognitive dysfunction in the aged canine. Neurobiol Learn Mem. 1996;66(1):11–23. 8661247 10.1006/nlme.1996.0039

[44] Head E, Pop V, Sarsoza F, et al Amyloid-β-peptide and oligomers in the brain and CSF of aged canines. J Alzheimers Dis. 2010;20(2):637–646. 20164551 10.3233/JAD-2010-1397 PMC2903832

[45] Gooch J, Wilcock DM. Animal models of vascular cognitive impairment and dementia (VCID). Cell Mol Neurobiol. 2016;36(2):233–239. 26988696 10.1007/s10571-015-0286-3 PMC11482509

[46] Kalaria RN . The pathology and pathophysiology of vascular dementia. Neuropharmacology. 2018;134(Pt B):226–239. 29273521 10.1016/j.neuropharm.2017.12.030

[47] Iadecola C, Duering M, Hachinski V, et al Vascular cognitive impairment in dementia: JACC scientific expert panel. J Am Coll Cardiol. 2019;73(25):3326–3344. 31248555 10.1016/j.jacc.2019.04.034 PMC6719789

[48] Vite CH, Head E. Aging in the canine and feline brain. Vet Clin North Am Small Anim Pract. 2014;44(6):1113–1129. 25441628 10.1016/j.cvsm.2014.07.008 PMC4254595

[49] Nakayama H, Uchida K, Doi K. A comparative study of age-related brain pathology—Are neurodegenerative diseases present in non-human animals? Med Hypotheses. 2004;63(2):198–202. 15236775 10.1016/j.mehy.2003.12.047

[50] Abey A, Davies D, Goldsbury C, Buckland M, Valenzuela M, Duncan T. Distribution of tau hyperphosphorylation in canine dementia resembles early Alzheimer’s disease and other tauopathies. Brain Pathol. 2021;31(1):144–162. 32810333 10.1111/bpa.12893 PMC8018065

[51] Bobik M, Thompson T, Russell MJ. Amyloid deposition in various breeds of dog. Neuroscience. 1994;20:172.

[52] Cory J . Identification and management of cognitive decline in companion animals and the comparisons with Alzheimer’s disease: A review. J Vet Behav. 2013;8(4):291–301.

[53] Gaillard F, Jayanti S, Rasuli B, et al Neurodegenerative MRI brain (an approach). Accessed 28 March 2023. Radiopaedia.org

[54] Karagiannis C, Mills D. Feline cognitive dysfunction syndrome. Vet Focus. 2014;24(2):42–47.

[55] Sordo L, Gunn-Moore DA. Cognitive dysfunction in cats: Update on neuropathological and behavioural changes plus clinical management. Vet Rec. 2021;188(1):e3. 34651755 10.1002/vetr.3

[56] Landsberg GM, Denenberg S, Araujo JA. Cognitive dysfunction in cats: A syndrome we used to dismiss as ‘old age’. J Feline Med Surg. 2010;12(11):837–848. 20974401 10.1016/j.jfms.2010.09.004 PMC11220932

[57] Černá P, Gardiner H, Sordo L, et al Potential causes of increased vocalisation in elderly cats with cognitive dysfunction syndrome as assessed by their owners. Animals (Basel). 2020;10(6):1092. 32599838 10.3390/ani10061092 PMC7341261

[58] Sordo L, Breheny C, Halls V, et al Prevalence of disease and age-related behavioural changes in cats: Past and present. Vet Sci. 2020;7(3):85. 32640581 10.3390/vetsci7030085 PMC7557453

[59] Cummings BJ, Satou T, Head E, et al Diffuse plaques contain C-terminal A beta 42 and not A beta 40: Evidence from cats and dogs. Neurobiol Aging. 1996;17(4):653–659. 8832640 10.1016/0197-4580(96)00062-0

[60] Sordo L, Martini AC, Houston EF, Head E, Gunn-Moore D. Neuropathology of aging in cats and its similarities to human Alzheimer’s disease. Front Aging. 2021;2:684607. 35822024 10.3389/fragi.2021.684607 PMC9261448

[61] Wippold FJ, Cairns N, Vo K, et al Neuropathology for the neuroradiologist: Plaques and tangles. AJNR Am J Neuroradiol. 2008;29(1):18–22. 17925367 10.3174/ajnr.A0781 PMC8119079

[62] Armstrong RA . A critical analysis of the ‘amyloid cascade hypothesis’. Folio Neuropathol. 2014;52(3):211–225. 25310732

[63] Gu J-I, Liu F. Tau in Alzheimer’s disease: Pathological alterations and an attractive therapeutic target. Curr Med Sci. 2020;40(6):1009–1021. 33428128 10.1007/s11596-020-2282-1

[64] Chambers JK, Tokuda T, Uchida K, et al The domestic cat as a natural animal model of Alzheimer’s disease. Acta Neuropathol Commun. 2015;3:78. 26651821 10.1186/s40478-015-0258-3 PMC4674944

[65] Hoffe B, Holahan MR. The use of pigs as a translational model for studying neurodegenerative diseases. Front Physiol. 2019;10:838. 31354509 10.3389/fphys.2019.00838 PMC6635594

[66] Arts JWM, van der Staay FJ, Ekkel ED. Working and reference memory of pigs in the spatial holeboard discrimination task. Behav Brain Res. 2009;205(1):303–306. 19539660 10.1016/j.bbr.2009.06.014

[67] Tanida H, Nagano Y. The ability of miniature pigs to discriminate between a stranger and their familiar handler. Appl Anim Behav Sci. 1998;56:149–159.

[68] Oddo S, Caccamo A, Shepherd JS, et al Triple-transgenic model of Alzheimer’s disease with plaques and tangles. Neuron. 2003;39(3):409–421. 12895417 10.1016/s0896-6273(03)00434-3

[69] Kragh PM, Nielson AL, Li J, et al Hemizygous minipigs produced by random gene insertion and handmade cloning express the Alzheimer’s disease-causing dominant mutation APPsw. Transgenic Res. 2009;18(4):545–558. 19184503 10.1007/s11248-009-9245-4

[70] Brubaker L, Udell MAR. Cognition and learning in horses (*Equus caballus*): What we know and why we should ask more. Behav Proccesses. 2016;126:121–131. 10.1016/j.beproc.2016.03.017 27018202

[71] Lampe JF, Andre J. Cross-modal recognition of human individuals in domestic horses (*Equus caballus*). Anim Cogn. 2012;15(4):623–630. 22526687 10.1007/s10071-012-0490-1

[72] Gray DA, Woulfe J. Lipofuscin and aging: A matter of toxic waste. Sci Aging Knowledge Environ. 2005;5:re1. 10.1126/sageke.2005.5.re1 15689603

[73] Sulzer D, Mosharov E, Talloczy Z, et al Neuronal pigmented autophagic vacuoles: Lipofuscin, neuromelanin, and ceroid as macroautophagic responses during aging and disease. J Neurochem. 2008;106(1):24–36. 18384642 10.1111/j.1471-4159.2008.05385.x PMC6609458

[74] Moreno-Garcia A, Kun A, Calero O, Medina M, Calero M. An overview of the role of lipofuscin in age-related neurodegeneration. Front Neurosci. 2018;12:464. 30026686 10.3389/fnins.2018.00464 PMC6041410

[75] Boellaard JW, Schole W, Hoefer W. Species-specific ultrastructure of neuronal lipofuscin in hippocampus and neocortex of subhuman mammals and humans. Ultrastruct Pathol. 2004;38(5–6):341–351. 10.1080/019131290882330 15764582

[76] Pugazhenthi S, Qin L, Hemachandra Reddy P. Common neurodegenerative pathways in obesity, diabetes, and Alzheimer’s disease. Biochem Biophys Acta. 2017;1863:1037–1045. 10.1016/j.bbadis.2016.04.017 PMC5344771 27156888

[77] Mendoza FJ, Toribio RE, Perez-Ecija A. Metabolic and endocrine insights in donkeys. Animals (Basel). 2024;14(4):590. 38396558 10.3390/ani14040590 PMC10885905

[78] Waragai M, Ho G, Takamatsu Y, et al Importance of adiponectin activity in the pathogenesis of Alzheimer’s disease. Ann Clin Transl Neurol. 2017;4(8):591–600. 28812049 10.1002/acn3.436 PMC5553221

[79] Costassa EV, Fiorini M, Zanusso G, et al Characterization of amyloid-β deposits in bovine brains. J Alzheimers Dis. 2016;51(3):875–887. 26890772 10.3233/JAD-151007 PMC4927890

[80] Reid SJ, Mckean NE, Henty K, et al Alzheimer’s disease markers in the aged sheep (*Ovis aries*). Neurobiol Aging. 2017;58:112–119. 28728117 10.1016/j.neurobiolaging.2017.06.020

[81] Davies ES, Morphew RM, Cutress D, et al Characterization of microtubule-associated protein tau isoforms and Alzheimer’s disease-like pathology in normal sheep (*Ovis aries*): Relevance to their potential as a model of Alzheimer’s disease. Cell Mol Life Sci. 2022;79(11):560. 36269420 10.1007/s00018-022-04572-z PMC9587068

[82] Hanggi EB, Ingersoll JF. Long-term memory for categories and concepts in horses (*Equus caballus*). Anim Cogn. 2009;12(3):451–462. 19148689 10.1007/s10071-008-0205-9

[83] Rausova P, Vochozkova P, Vidinska D, et al Porcine model of Huntington’s disease. Huntington’s disease—Molecular pathogenesis and current models. InTech; 2017.

[84] Jacobsen JC, Bawden CS, Rudiger SR, et al An ovine transgenic Huntington’s disease model. Hum Mol Genet. 2010;19(10):1873–1882. 20154343 10.1093/hmg/ddq063 PMC2860888

[85] Sacchini S, Delgado-Diaz J, Espinosa de los Monteros A, et al Amyloid-beta peptide and phosphorylated tau in the frontopolar cerebral cortex and in the cerebellum of toothed whales: Aging versus hypoxia. Biol Open. 2020;9(11):bio54734. 10.1242/bio.054734 PMC7657478 33037014

[86] Bearzi G, Reggente MAL. Epimeletic behavior. Encycl Mar Mamm. 2018;3:337–338.

[87] Scottish Marine Animal Stranding Scheme (SMASS). Map of Strandings . Accessed 21 March 2023. https://strandings.org

[88] Oelschläger HHA . The dolphin brain—A challenge for synthetic neurobiology. Brain Res Bull. 2008;75(2–4):450–459. 18331914 10.1016/j.brainresbull.2007.10.051

[89] Cozzi B, Huggenberger S, Oelschläger H. Anatomy of dolphins: Insights into body structure and function. Elsevier; 2017.

[90] Braak H, Braak E. Neuropathological stageing of Alzheimer-related changes. Acta Neuropathol. 1991;82(4):239–259. 1759558 10.1007/BF00308809

[91] Pereyra G, Bovolenta P. Of dolphins, humans, other long-lived animals and Alzheimer’s disease (commentary on Vacher et al.). Eur J Neurosci. 2023;57:1180–1183. 36861213 10.1111/ejn.15946

[92] Stylianaki I, Komnenou AT, Posantzis D, et al Alzheimer’s disease-like pathological lesions in an aged bottlenose dolphin (*Tursiops truncatus*). Vet Rec Case Rep. 2019;7(1):e000700.

[93] Ashok BS, Ajith TA, Sivanesan S. Hypoxia-inducible factors as neuroprotective agent in Alzheimer’s disease. Clin Exp Pharmacol Physiol. 2017;9(11):327–334. 10.1111/1440-1681.12717 28004401

[94] Perl DP . Neuropathology of Alzheimer’s disease. Mt Sinai J Med. 2010;77(1):32–42. 20101720 10.1002/msj.20157 PMC2918894

[95] Mandelkow EM, Mandelkow E. Biochemistry and cell biology of tau protein in neurofibrillary degeneration. Cold Spring Harb Perspect Med. 2012;2(7):a006247. 22762014 10.1101/cshperspect.a006247 PMC3385935

[96] Liu W, Wong A, Law ACK, Mok VCT. Cerebrovascular disease, amyloid plaques and dementia. Stroke. 2015;46(5):1402–1407. 25765727 10.1161/STROKEAHA.114.006571

[97] Xiong M, Jiang H, Serrano JR, et al APOE immunotherapy reduces cerebral amyloid angiopathy and amyloid plaques while improving cerebrovascular function. Sci Transl Med. 2021;13(581):eabd7522. 33597265 10.1126/scitranslmed.abd7522 PMC8128342

[98] Gorman AM . Neuronal cell death in neurodegenerative diseases: Recurring themes around protein handling. J Cell Mol Med. 2008;12(6A):2263–2280. 18624755 10.1111/j.1582-4934.2008.00402.x PMC4514105

[99] Kim J, Eltorai AEM, Jiang H, et al Anti-apoE immunotherapy inhibits amyloid accumulation in a transgenic mouse model of Aβ amyloidosis. J Exp Med. 2012;209(12):2149–2156. 23129750 10.1084/jem.20121274 PMC3501350

[100] Wu M, Chen Z, Jiang M, et al Friend or foe: Role of pathological tau in neuronal death. Mol Psychiatry. 2023;28(6):2215–2227. 36918705 10.1038/s41380-023-02024-z

[101] DeKosky ST, Scheff SW. Synapse loss in frontal cortex biopsies in Alzheimer’s disease: Correlation with cognitive severity. Ann Neurol. 1990;27(5):457–464. 2360787 10.1002/ana.410270502

[102] Scheff SW, Price DA, Schmitt FA, DeKosky ST, Mufson EJ. Synaptic alterations in CA1 in mild Alzheimer disease and mild cognitive impairment. Neurology. 2007;68(18):1501–1508. 17470753 10.1212/01.wnl.0000260698.46517.8f

[103] Scheff SW, Price DA, Schmitt FA, Mufson EJ. Hippocampal synaptic loss in early Alzheimer’s disease and mild cognitive impairment. Neurobiol Aging. 2006;27(10):1372–1384. 16289476 10.1016/j.neurobiolaging.2005.09.012

[104] Terry RD, Masliah E, Salmon DP, et al Physical basis of cognitive alterations in Alzheimer’s disease: Synapse loss is the major correlate of cognitive impairment. Ann Neurol. 1991;30(4):572–580. 1789684 10.1002/ana.410300410

[105] Animal Welfare Act . Accessed 03 April 2023. https://www.legislation.gov.uk/ukpga/2006/45/contents

[106] Legislation.gov.uk Animals (Scientific Procedures) Act 1986 . Accessed 23 November 2024. https://www.legislation.gov.uk/ukpga/1986/14/section/1

[107] World Wildlife Fund (WWF). 10 of the world's most endangered animals . Accessed 03 April 2023. https://www.wwf.org.uk/learn/wildlife/endangered-animals

